# Nicolau Syndrome Following Glatiramer Injection in a Middle-Aged Woman

**DOI:** 10.7759/cureus.53746

**Published:** 2024-02-06

**Authors:** Gun Min Youn, Karan S Cheema, Peter A Young, Gordon H Bae

**Affiliations:** 1 Department of Dermatology, Stanford University School of Medicine, Palo Alto, USA; 2 School of Public Health, Brown University, Providence, USA; 3 Department of Dermatology, Kaiser Permanente, Sacramento, USA

**Keywords:** embolia cutis medicamentosa, glatiramer, patients with multiple sclerosis, livedo like dermatitis, nicolau syndrome

## Abstract

Nicolau syndrome is a rare adverse reaction that can occur in the setting of intramuscular, intravenous, and subcutaneous injections. Proper diagnosis and management are critical to avoid complications including abscesses, muscular atrophy, and necrotizing fasciitis. Here, we report a 55-year-old female with multiple sclerosis who presented to our clinic following a subcutaneous injection of 40mg of glatiramer. She immediately noted a sharp pain and erythema, which developed into a purple discoloration, became purulent, and eventually necrosed. The patient’s wound was debrided, and she was advised to clean the wound with soap and water, apply topical mupirocin, and change dressings twice daily. She continued to receive appropriate follow-up care with weekly to bi-weekly debridement with excellent resolution.

## Introduction

Nicolau syndrome (NS), also known as livedo-like dermatitis or embolia cutis medicamentosa, is a rare adverse injection reaction that can present following subcutaneous, intramuscular, or intravenous injections [1,2]. This condition has been linked to a variety of medications including local anesthetics, benzathine penicillin, corticosteroids, and immunomodulatory drugs such as tumor necrosis factor-alpha inhibitors [3].

NS classically unfolds as immediate pain and erythema at an injection site, which becomes a livedoid violaceous patch, followed by localized necrosis [2]. Progression is difficult to predict and complications may include abscess, muscular atrophy, myositis, and rarely necrotizing fasciitis [1,2]. Once symptom resolution occurs, recurrence of NS from the same medication is extremely rare, but instances of it have been reported in the literature [4]. Here, we report a case of NS following a subcutaneous injection of glatiramer with images at the time of presentation and resolution to visually demonstrate this rare condition.

## Case presentation

**Figure 1:**
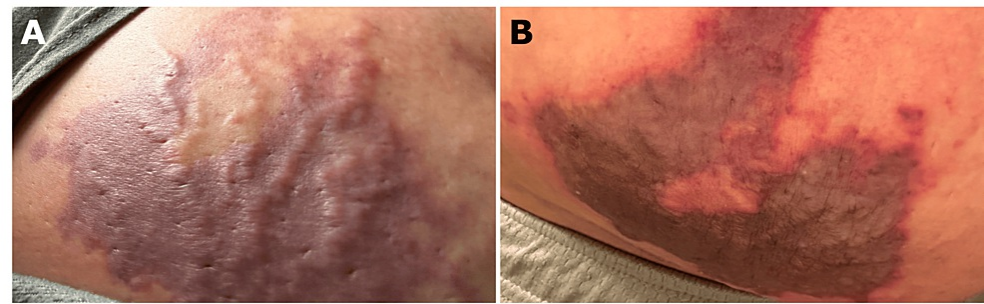
(A) Purple plaque three days post-injection with glatiramer. (B) Purple plaque with evidence of skin necrosis 10 days post-injection.

A 55-year-old female with a history of multiple sclerosis on glatiramer presented with a necrotic reticulated plaque on her abdomen. She reported having severe pain immediately post-injection of glatiramer, followed by patchy redness which quickly spread to adjacent skin. By the third day, the patch had developed into a purple plaque (Figure 1A) with evidence of skin necrosis by the 10th post-injection day (Figure 1B). She denied a history of adverse reactions during the several years she had been self-administering glatiramer 40 mg three times weekly.

Physical examination revealed a well-demarcated 8 x 10 cm brown-to-black geometric reticulated plaque on the right lower abdomen (Figure 1A). As there is no conclusive test for NS, the diagnosis is a clinical one that must take into account the patient’s history, presentation, and symptoms after an injection. Our differential diagnosis included necrotizing fasciitis, cellulitis, gas gangrene, fat embolism, toxic drug reaction, and nerve injury. In our patient, the clinical history of immediate injection site pain and discoloration with no infectious symptoms or findings led to our diagnosis of NS.

**Figure 2:**
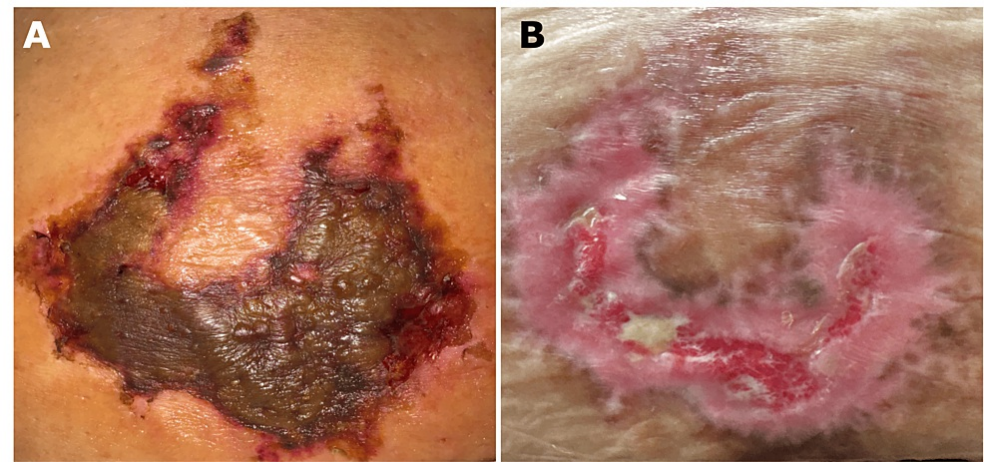
(A) Black to brown reticulated plaque 18 days post-injection. (B) Significant re-epithelization with minimal residual granulation tissue, 54 days post-injection.

The lesion was debrided, and the patient was advised to clean the wound with soap and water, apply topical mupirocin, and change dressings twice daily. The patient received six weeks of follow-up care for weekly to bi-weekly debridement of necrotic tissue and wound monitoring with resolution of her lesion (Figures 2A, 2B).

## Discussion

NS was first described in the early 20th century in the setting of intramuscular bismuth salt injections for syphilis treatment [5]. Since then, reports of this syndrome have been documented following subcutaneous, intravenous, and even intraarticular injections. However, literature on NS secondary to agents such as subcutaneous glatiramer, as demonstrated in our report, remains relatively sparse [5]. Thus, we report a rare case of subcutaneous glatiramer injection-induced NS, its progression, treatment, and resolution.

While there are several theories on the pathogenesis of NS, the exact mechanism remains unclear. Several experiments have ruled out immunologic reactions, injection speed, drug properties, and microbial contaminants as instigators [1,3]. The prevailing theory is that iatrogenic arterial complications lead to occlusion of vascular flow and necrosis within the arterial distribution. One potential mechanism for this is arterial or peri-arterial injection, leading to significant inflammation that destroys the artery, cutting off vascular flow [5]. This was demonstrated in an early rabbit model by Brachtel and Meinertz, who found that intraarterial and peri-arterial injections led to cutaneous necrosis with severe arterial inflammation and destruction upon histological analysis [6]. Other possible mechanisms include vasospasm or thrombi, resulting in impaired blood flow and necrosis, with the latter of these hypotheses supported by histologic results demonstrating the presence of micro-emboli within the arterial supply [1,5].

There is no standard treatment for NS given its rarity, though close management is crucial to ensure resolution and prevent infection. Current reported treatments include heparin and topical or systemic corticosteroids due to their vasoactive properties, topical antibiotic ointments for infection prophylaxis, and surgical debridement [1-3]. Additionally, in patients experiencing significant pain, appropriate analgesics should be prescribed for symptomatic management. In our case, given the extent of necrosis, we opted for debridement as well as topical antibiotics for infection prophylaxis. Proper wound care with soap and water as well as regular dressing changes were also emphasized to mitigate the risk of further complications. While our patient responded well to our treatment regimen, further work should explore pathways that may expedite resolution while minimizing complications.

Despite the low probability of developing post-injection NS, given the morbidity associated with this condition, proper steps should be taken to minimize risk. In 2022, Mojarrad et al. noted that proper injection site choice as well as utilizing appropriately sized needles could potentially reduce the risk of NS development [5]. Particular emphasis was placed on these preventative measures to avoid vascular structures, given the hypothesis that NS can be caused by intra/peri-arterial injections. Additionally, as noted by Lie et al., the Z-track method, which de-aligns the skin and underlying muscle by pulling the skin perpendicular to the needle can also be used, as this is hypothesized to break the needle track and minimize subcutaneous irritation [7,8]. The risk of NS can also be further minimized by aspirating prior to injection, not administering more than 5mL of medications per site, and changing sites if more than 5mL of medication is required per administration [9]. Thus, we urge clinicians to both fully educate patients such as ours with regular self-injections, and to also be aware of NS as a possible complication that should be rapidly treated to prevent further complications.

## Conclusions

NS is a rare adverse injection reaction that can occur in the setting of self-injection of immunomodulatory drugs such as glatiramer. As demonstrated by our case, physicians should have a high index of suspicion when patients on glatiramer present with immediate post-injection pain and erythema which progresses to necrosis. Additionally, as evidenced in our case study, the combination of appropriate wound care with topical antibiotics and regular dressing changes, along with weekly to bi-weekly debridement, was able to provide excellent resolution in this patient with NS and can potentially be used for other patients with this condition.
